# In-depth Investigation of Hg_2_Br_2_ Crystal Growth and Evolution

**DOI:** 10.3390/ma12244224

**Published:** 2019-12-16

**Authors:** Tae Hyeon Kim, Hee Tae Lee, Young-Min Kang, Gun-Eik Jang, In Hoi Kwon, Byungjin Cho

**Affiliations:** 1 Department of Advanced Material Engineering, Chungbuk National University, Chungbuk 28644, Korea; kth2595@naver.com (T.H.K.); gejang@chungbuk.ac.kr (G.-E.J.); 2 Optical Material Group, Green Optics, Chungbuk 28126, Korea; leeht@greenoptics.com (H.T.L.); youngmin3596@greenoptics.com (Y.-M.K.)

**Keywords:** Hg_2_Br_2_, PVT, seed, crystal growth

## Abstract

Physical vapor transport (PVT) has frequently been adopted for the synthesis of mercurous bromide (Hg_2_Br_2_) single crystals for acousto-optic modulators. However, thus far, very few in-depth studies have been conducted that elucidate the growth process of the Hg_2_Br_2_ single crystal. This paper reports an in-depth investigation regarding the crystal growth and evolution behavior of the Hg_2_Br_2_ crystal with facet growth mode. Based on the experimental and simulation results, the temperature profile conditions concerning the seed generation and seed growth could be optimized. Next, the PVT-grown Hg_2_Br_2_ crystals (divided into single crystal and quasi-single crystal regions) were characterized using various analysis techniques. The single-crystal Hg_2_Br_2_ was found to possess a more uniform strain than that of the quasi-single crystal through a comparison of the X-ray diffraction data. Meanwhile, the binding energy states and electron backscatter diffraction images of the as-synthesized Hg_2_Br_2_ crystals were similar, regardless of the crystal type. Furthermore, Raman spectroscopy and transmission electron microscopy analyses provided information on the atomic vibration mode and atomic structures of the two kinds of samples. The synergistic combination of the simulation and experimental results used to verify the growth mechanism facilitates the synthesis of high-quality Hg_2_Br_2_ crystals for potential acousto-optic tunable filter device applications.

## 1. Introduction

Acousto-optic tunable filters (AOTFs) have attracted a great deal of attention as a core module of ultra-spectral image sensing systems. They comprise piezoelectric transducers and acousto-optic modulator (AOM) single crystals combined together. AOTF devices utilize the principle of changing and decomposing the frequency of light using soundwaves. The vibration of the piezoelectric transducer changes the diffraction characteristics of the light incident on the AOM material, which in turn only diffracts light of a certain wavelength. Thus, the unique acousto-optical properties allow the AOTF to selectively obtain a diffraction signal for each wavelength by modulating the frequency applied to the AOTF apparatus. AOTF devices have potential use for a variety of applications, such as measurements of a cell monolayer, mineral exploration, environmental monitoring, process control, and the identification of toxic biological agents [1,2,3,4,5].

Specifically, toxic aerosols can be selectively identified, which enables superior performance in remote identification, detection, and tracking. Because the emission/absorption spectra corresponding to chemical agents and toxic substances are primarily found in the infrared region, with wavelengths of 8–12 μm, AOM crystal materials covering this range should be explored [1,3,6,7,8]. Meanwhile, the figure of merit (***M***_2_) index for evaluation of the optical properties of an AOM material is generally expressed by the following Equation:

(1) $$M_{2}=\frac{n_{i}^{3}n_{d}^{3}p^{2}}{\rho V^{3}}$$

where *n_i_* is the refractive index of the incident light; *n_d_* is the refractive index of the diffracted light; p is the effective photoelastic coefficient; ρ is the density of the AOM material; and *V* is the speed of sound [1]. Strong candidates for use as AOM materials include tellurium (Te), thallium arsenic selenide (Tl_3_AsSe_3_ or TAS), and mercurous halides (Hg_2_Cl_2_ and Hg_2_Br_2_) [2,4]. Although Te has the highest performance index (*M*_2_) among these, it is easily activated by heat because of its small bandgap and relatively weak mechanical strength. Meanwhile, TAS is a very toxic material, making it difficult to handle, thus requiring large facility construction costs [9]. However, the mercurous bromide (Hg_2_Br_2_) single-crystal material has advantages including a relatively wide optical transmission band (0.4–30 μm), wide birefringence, high nonlinear optical properties, and a high refractive index. Its figure of merit is also 2.5 times larger than that of the Hg_2_Cl_2_ single crystal with a similar lattice structure [10,11,12]. Furthermore, this material has the unique characteristic of being easily decomposed into gas phases of Hg and HgBr_2_ before melting. The physical properties make the physical vapor transport (PVT) process of the Hg_2_Br_2_ suitable [1]. In this process, it is important to grow a high-quality AOM single crystal that can be applied to the fabrication of a high-performance AOTF device. For example, Kim et al. have reported the study showing the achievement of excellent AOTF performance with the high-quality AOM single crystals [1,3,4,13]. However, most of the corresponding research has focused on the integrated AOTF system rather than AOM crystal growth. There are very few studies that investigate the growth and evolution mechanism of the Hg_2_Br_2_ crystal in-depth, which is also essential for the realization of a high-performance AOTF system.

Herein, we investigate the crystal growth and evolution behavior of the Hg_2_Br_2_ crystal synthesized using PVT. The temperature profile conditions of the seed growth were optimized from the comparison between the experimental and simulation results. Two types of PVT-grown Hg_2_Br_2_ crystals (single and quasi-single) were characterized using various analysis techniques. The single-crystal Hg_2_Br_2_ offered a more uniform strain than that of the quasi-single crystal, as verified by X-ray diffraction (XRD). Meanwhile, the binding energy states of the as-synthesized Hg_2_Br_2_ crystals were similar regardless of the crystal type. Furthermore, Raman spectroscopy and transmission electron microscopy (TEM) analyses provided the comparison results concerning the atomic vibration mode and atomic structures of the two kinds of samples. Thus, this in-depth investigation of the growth mechanism of the Hg_2_Br_2_ crystals introduces a way toward potential acousto-optic tunable filter device applications.

## 2. Materials and Methods

### 2.1. Purification Process of Hg_2_Br_2_ Powders

Prior to the growth of the Hg_2_Br_2_ single crystals, original Hg_2_Br_2_ powders were purified. To remove unintentional impurities adsorbed on the inner surface of the quartz ampoule, it was cleaned with high-purity acetone (99.99%), isopropyl alcohol (99.999%), and ultrapure water (99.999%). After this, the ampoule was placed into a furnace with four heating zones, for precise control of the temperature in each section, and dried at 150 °C for 1 h at a pressure of 10^−3^ torr. The Hg_2_Br_2_ (99.9%) powder was then loaded to the precleaned ampoule and dried at 100 °C for 1 h at 10^−5^ torr to completely remove the adsorbed moisture in the ampoule. The purification process of the powder was conducted by PVT at 300 °C for 10 h under the vacuum sealing condition and then the chamber was slowly cooled at a rate of 1 °C/min to prevent the formation of Hg metal or the corresponding secondary phases. The finally purified Hg_2_Br_2_ powder showed an extremely high 5 N purity of 99.999%, which was analyzed by inductively coupled plasma atomic emission spectroscopy, as reported in the previous work [14].

### 2.2. Growth Process of Hg_2_Br_2_ Crystals

The pre-purified Hg_2_Br_2_ material (99.999%) was charged into a quartz ampoule for the crystal growth, after which it was sealed under high vacuum conditions of 10^−6^ torr. To agglomerate the raw materials before crystal growth, a bottom heater was first heated to 330 °C and an upper heater was used to maintain a temperature gradient of 20 °C lower than the bottom heater. This first transfer process of Hg_2_Br_2_ raw powder was essential in the vertical type PVT system, condensing the sublimated Hg_2_Br_2_ powder on the uppermost region of the quartz ampoule [1]. In this step, the solid Hg_2_Br_2_ is dissociated into gas Hg and HgBr_2_ and then solidified again with the Hg_2_Br_2_. The actual Hg_2_Br_2_ crystal growth could be divided into three main processes: the formation and growth of the seed crystal, and the crystal growth. First, the seed crystal formation could be optimized by varying the temperature gradient of the heater. Second, it should be noted that, after seed formation, it is necessary to lower the temperature near the seed region for continuous crystal growth. In our case, the seed growth was governed by two main processing factors: controlling the temperature of the bottom heater and changing the position of the ampoule. Finally, the crystal evolved with the growth rate to a speed range of 2–5 mm/day. The cooling process was conducted for several days. The resulting grown crystal had a diameter of ~35 mm and a length of 70–80 mm. The simulation results were obtained using a Virtual Reactor simulator purchased from STR group, Inc (Saint Petersburg, Russia).

### 2.3. Characterization of Hg_2_Br_2_ Crystal

The grown crystals were basically characterized by X-ray photoelectron spectroscopy (XPS, PHI Quantera-II; by Ulvac-PHI (Osaka, Japan)) and XRD (X’Pert PRO MRD by PANAnalytical (Seong-Nam, Korea)) to identify the stoichiometric Hg_2_Br_2_ compound. In-depth analysis methods, including Raman spectroscopy, electron backscatter diffraction (EBSD, S-4800 by Hitachi (Seoul, Korea)), and TEM (TitanTM 80-300 by Titan (Hillsboro, OR, USA)), were adopted to study the atomic vibration modes, macroscopic crystallinity, and atomic structures. In the Raman spectroscopy, the polarized 532 nm radiation of a green laser (FEX by NOST (Seong-Nam, Korea)) was used as an excitation source. We prepared a TEM specimen using a special cryogenic-focused ion beam (FIB, Quanta 3D FEG by FEI (Hillsboro, OR, USA)) system, which was then observed. In particular, to minimize the sample damage occurring during the FIB process, the TEM sample was made under cryogenic conditions (i.e., at 63 K). When the TEM sample reached room temperature after the FIB processing was complete, the prepared specimens were moved to the TEM equipment to minimize sample damage using a vacuum transfer process. All the measurements were carried out at room temperature.

## 3. Results and Discussion

**Figure 1:**
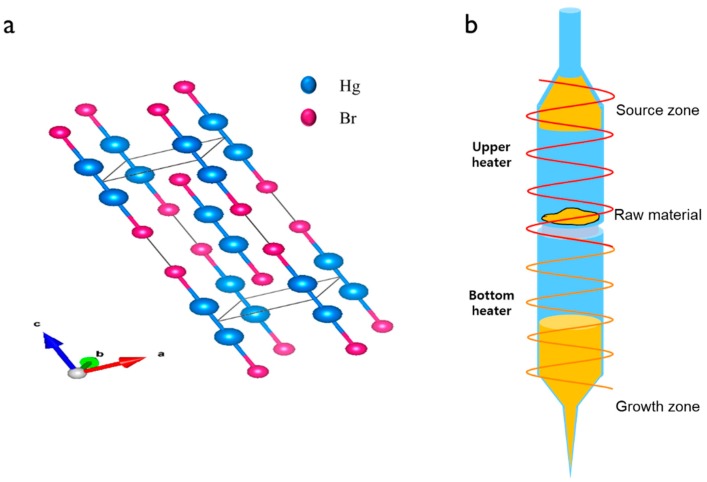
(**a**) Lattice structure of Hg_2_Br_2_. (**b**) Schematic of a quartz ampoule showing the source, raw material, and growth zones.

Figure 1a shows the tetragonal lattice structure of the Hg_2_Br_2_ crystal with a space group of I4/mmm (139), which comprises strong covalent bonding along the *c*-axis and relatively weak van der Waals bonds between the adjacent molecules along the *ab*-plane [15]. The lattice parameters a and c of Hg_2_Br_2_ are 4.65 and 11.10 Å, respectively [12,13]. This unique atomic structure causes an optically anisotropic property. The PVT process was selected to grow the Hg_2_Br_2_ single crystal because Hg_2_Br_2_ material can be sublimated at relatively low temperatures [15,16]. The PVT system designed for the Hg_2_Br_2_ crystal growth comprised three parts: the control unit for manipulating the temperature/time, the driving unit for moving the ampoule, and the chamber/heater unit that surrounded the ampoule. The ampoule partly surrounded by the heater could be divided into three zones: source, raw material, and growth zone (Figure 1b). The pre-purified Hg_2_Br_2_ powder, prepared by a PVT-based purification process, was loaded on a ceramic membrane filter located in the center of the ampoule, for the passing of only small Hg_2_Br_2_ particles (<150 μm) to the bottom growth zone of the ampoule. The lower part of the growth zone had a conical-shaped tip while the upper source zone part had a cylindrical shape.

**Figure 2:**
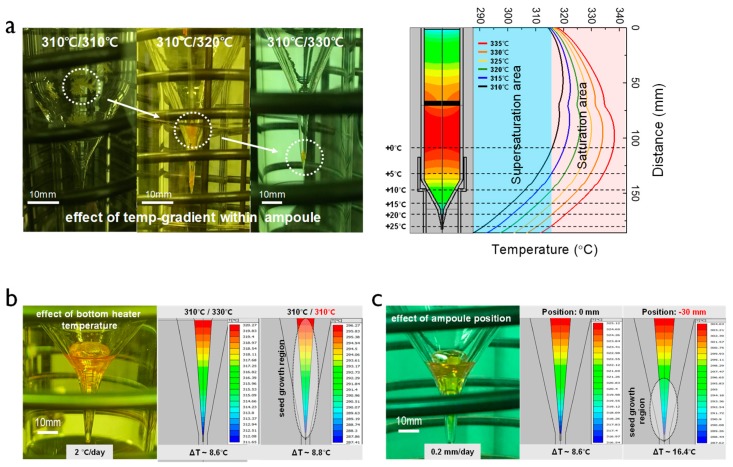
(**a**) Experiment (left) and simulation (right) for seed generation modulated by the temperature gradient within the ampoule. Effect of two processing parameters on seed growth of Hg_2_Br_2_: (**b**) bottom heater temperature and (**c**) ampoule position. The actual experiments and simulation are compared in the two latter cases.

We checked the effects of the temperature gradient within the ampoule, the bottom heater temperature, and the ampoule position to explore the optimization condition of the formation and growth of the seed crystal. First, the different temperature gradient profiles within the ampoule were obtained experimentally and compared with the simulation results (Figure 2a). When the temperature of the bottom heater was changed from 310 °C to 330 °C at a fixed upper heater temperature of 310 °C, different seed formation behaviors were obtained throughout the ampoule region. The experimental results show that the initial Hg_2_Br_2_ seed was observed in the upper wall of the ampoule at the temperature gradient of 0 °C. However, the seed was generated at the end of the tip for the temperature gradient of 20 °C, which was preferred for stable crystal growth thereafter. The simulation results show that Hg_2_Br_2_ nucleation occurred in a supersaturation area (light blue region in Figure 2b). More specifically, as the temperature of the heater increased from 310 to 335 °C, the supersaturation area decreased (i.e., overlap between the supersaturation area and temperature profile decreased with increasing temperature of the heater). These results indicate that the temperature gradient profile determines the position of the initial seed formation. The simulation result is also highly consistent with the experimental result.

Secondly, we explored the effect of the bottom heater temperature on the step of crystal growth after the formation step of the seed, as shown in Figure 2b. The rate of lowering the temperature of the bottom heater was carried out at 2 °C/day. We confirmed that the reduction of the bottom heater temperature caused the crystal including single- and polycrystalline crystal to have randomly oriented facets, as confirmed by the optical image of the crystal with opaque color (left of Figure 2b). When the bottom heater temperature decreased from 330 to 310 °C in the simulation (right of Figure 2b), the overall temperature of the seed portion decreased, but the temperature difference changed negligible (i.e., the total temperature of the seed portion was uniformly lowered). These results confirm that nucleation sites occurred on a relatively wide region of the wall, thereby facilitating polycrystalline growth. Finally, the effect of the ampoule position on the crystal growth mode was investigated (Figure 2c). The ampoule position was lowered by 30 mm, and the descent speed of the ampoule was 2 mm/day. As validated by the optical image (left of Figure 2c), a single crystal phase with high transparency was preferably formed by lowering the position of the ampoule by 30 mm under fixed temperatures of the heaters. In the simulation (right of Figure 2c), the temperature difference was changed from 8.6 to 16.4 °C by lowering the ampoule position, which was more effective for causing a high temperature difference. Such an abrupt temperature difference of the seed crystal confines the region of the crystal growth to the end of the ampoule tip, narrowing the nucleation boundary and thereby leading to a stable growth mode.

**Figure 3:**
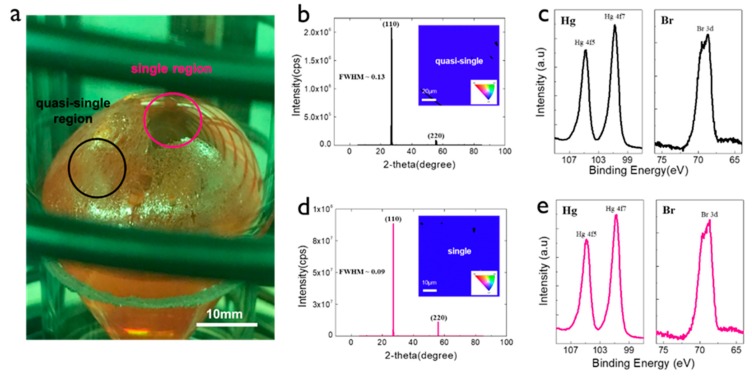
(**a**) Optical image of as-grown Hg_2_Br_2_ crystal with single and quasi-crystal regions; (**b**) XRD and (**c**) XPS spectra of quasi-single crystal region; (**d**) XRD and (**e**) XPS spectra of single crystal region. The insets of (**b**,**d**) show the EBSD mapping image of the corresponding regions.

We adopted the ampoule position change described above to grow the Hg_2_Br_2_ crystal. Figure 3a displays an optical image of the as-grown Hg_2_Br_2_ crystal with two different growth regions (center and edge of the crystal) showing transparent and opaque colors (denoted as the single and quasi-single crystals). The XRD peak data of the Hg_2_Br_2_ samples exhibited the primary (110) and its family (220) planes, regardless of the kind of extracted sample (Figure 3b,d) [17]. The 2θ positions of the major planes in the two samples (crystal and quasi-crystal) were similar. Nevertheless, the single Hg_2_Br_2_ crystal exhibited relatively higher peak intensities for all planes, indicating more perfect crystallinity. In general, the full width at half maximum (FWHM) values in θ/2θ XRD measurements usually correspond to lattice distortion [18]. The FWHM values of the (110) planes in the quasi-single and single crystals were calculated to be 0.13 and 0.09, respectively. The FWHM values usually correspond to lattice distortion. It is highly likely that a crystal with nonuniform strain in the lattice makes the FWHM comparatively broad. In our grown Hg_2_Br_2_ crystal, the FWHM of the single crystal was 1.4 times lower than that of the quasi-single crystal, which indicates a better crystal with no lattice distortion. The insets of Figure 3b,d show the EBSD mapping results on the quasi-single and single crystals, showing a single Hg_2_Br_2_ crystal stacked with the (110) plane. This is strongly consistent with the XRD results. To identify the chemical compound states, the XPS data of the two crystals were obtained, showing that the peaks of Hg 4f5, 4f7, and Br 3d are exactly the same as the XPS data of the purified Hg_2_Br_2_ powder measured in the previous study [19,20]. The binding energies of Hg 4f7 and Br 3d in the quasi-single crystal were 100.8 eV and 68.9 eV, respectively, and the peak positions of the single crystal were observed at 100.7 eV and 68.7 eV. There was no remarkable difference in the XPS peak positions for the two samples. Even if there was lattice distortion in the quasi-single crystal, the chemical compound states remained the same regardless of the growth region.

**Figure 4:**
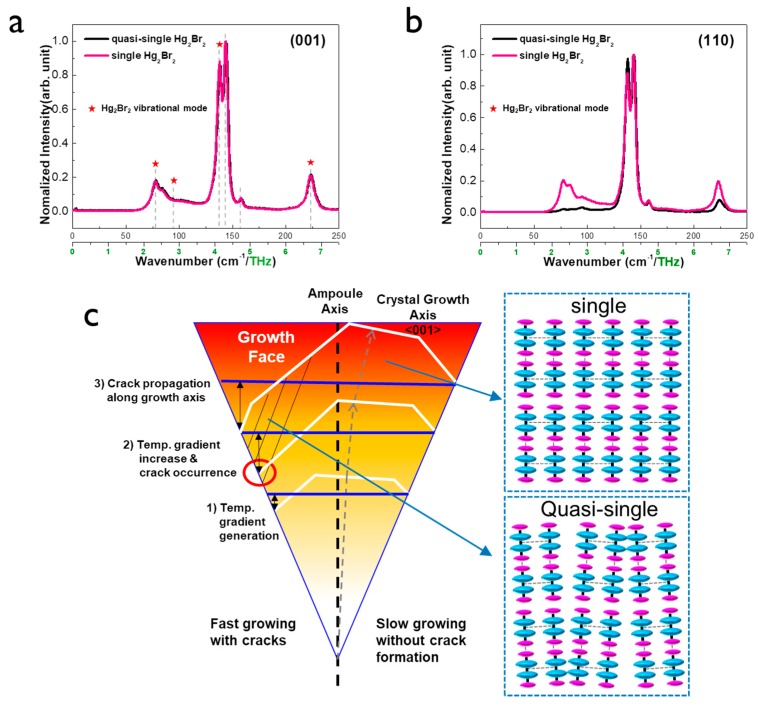
Comparison of Raman spectra according to quasi-single and single Hg_2_Br_2_ crystals for (**a**) (001) and (**b**) (110) planes; (**c**) Schematic of proposed Hg_2_Br_2_ crystal growth mechanism and the expected bonding structures of the single and quasi-single crystals.

To check the atomic vibration mode of the two crystals, Raman spectroscopy analysis was performed along the crystal plane direction (Figure 4a,b). In the vibrational spectra of Hg_2_Br_2_, the four prominent peaks (35.5, 91, 136, and 221 cm^−1^) follow the group theory of the selection rules: the two fully symmetric peaks with A_1g_ symmetry and two doubly degenerated peaks with E_g_ symmetry modes. These symmetry modes indicate low-frequency vibrations (35.5 and 91 cm^−1^) and high-frequency vibrations (136 and 221 cm^−1^), respectively. The first vibration mode of E_g_ symmetry (35.5 cm^−1^) is the librations of the linear molecules as a whole with respect to *Z*-axis, while the second vibration of E_g_ symmetry (91 cm^−1^) is the deformed zigzag ones. Meanwhile, the fully symmetric valence vibration A_1g_ mainly corresponds to Hg–Hg (136 cm^−1^) and Br–Hg (221 cm^−1^) displacements [21,22]. Information about the peak position of Hg_2_Br_2_ obtained experimentally and theoretically is described in Table S1 in the supporting information. Overall, the peaks involving the vibration modes of Hg_2_Br_2_ (featured as star mark) were clearly observed, while extra peaks were detected as well, which are not consistent with the conventional Hg_2_Br_2_. Unfortunately, no Raman database can match the extra peaks. The origin of the corresponding extra peaks should be further explored.

Overall, the peaks of the Hg_2_Br_2_ vibrational mode on the (001) plane appeared almost the same regardless of a kind of crystal (quasi-single or single) as shown in Figure 4a. There was no significant difference in Raman spectra between quasi-single and single Hg_2_Br_2_ crystals. The first vibration peak was negligible due to the resolution limit of Raman analysis equipment. However, the peaks involving the second and fourth vibration modes in single crystal showed higher intensity than that of quasi-single when the information of the Raman spectra was collected on the (110) plane. It is obvious that there was no strong plane dependency in the Raman spectra of the single crystal while the quasi-single crystal showed the interesting result of the Raman spectra strongly depending on the crystal plane. It might be due to the slight difference in van der Waals interaction in the intermolecular structure (right of Figure 4c). Especially, quasi-single crystal has less intermolecular interaction due to the imperfect columnar-shaped structure of Hg_2_Br_2_, causing less vibration. For more detailed polarized vibrational analyses, we performed Raman mapping analysis on the large area of 1400 × 1400 μm^2^ (Figure S1 in supporting information). The large area mapping result was highly consistent with point data. Based on all the experimental and simulation results, the proposed model of the Hg_2_Br_2_ crystal growth illustrates the evolution of the crystal facets in the center and wall of the ampoule (Figure 4c). It is reasonably acceptable that the Hg_2_Br_2_ crystal does not appear to grow in the perfectly vertical direction of the ampoule, but rather in a slightly tilted direction [13]. When the Hg_2_Br_2_ phase was grown, individual faceted crystals were formed but the facet on the (001) plane was most preferable. This tends to be similar to (0001) facet growth of SiC based on the same PVT process [23]. The crystal growth along the <001> direction is usually more preferable than that along the <110> direction [24]. This occurs to reduce the total surface energy of the material system. A temperature gradient between the ampoule wall and growth surface of the Hg_2_Br_2_ crystal can be generated (red circle of Figure 4b), causing cracks near the ampoule. These cracks expand toward the parallel direction of crystal growth with increasing time. Weak van der Waals bonds between adjacent Hg_2_Br_2_ molecules can facilitate the crack propagation, thus forming a quasi-single crystal with a slightly tilted columnar-shaped Hg_2_Br_2_ structure, unlike the perfect alignment of the Hg_2_Br_2_ lattice on the single crystal.

**Figure 5:**
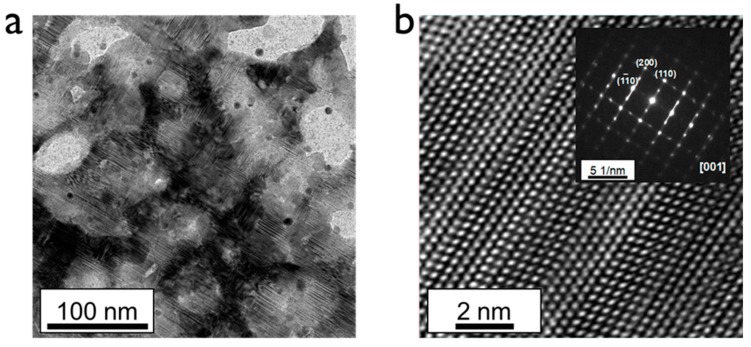
(**a**) Low-magnified TEM and (**b**) high-magnified atomic resolution TEM images of single-crystalline Hg_2_Br_2_. The inset of (**b**) shows the corresponding electron diffraction image.

To verify the interatomic arrangements and lattice structures of the two samples, TEM measurements were performed. Figure 5a shows a low-magnified TEM image of a single Hg_2_Br_2_ crystal. Layered atomic structures were clearly observed due to the unique lattice comprising covalent and van der Waals bonding. Meanwhile, the black region shows the intrinsic single-crystal region and the bright-colored region shows the empty region structurally collapsed. The highly magnified TEM image clearly shows the atomic arrangement of the Hg and Br elements (Figure 5b). The inset of Figure 5b, a selected area electron diffraction (SAED) pattern along the [001] zone axis, exhibits typical periodic dot patterns of [(110), (200), and (1 $\bar{1}$ 0)], representing a tetragonal structure [25]. As shown in the TEM image of a single crystal, many stacking faults appeared due to the layer-type structure with relatively weak van der Waals bonds. Especially, more stacking faults existed and were propagated over the whole region of quasi-single crystals with relatively imperfect intermolecular bonding structure, causing irrecoverable damage on the preparation process of TEM specimen (Figure S2 in supporting information). This might be because the symmetry was easily broken by the stacking faults and many phonons from all Brillouin zones became active [26]. Thus, the structure of the quasi-single crystal sample was unstable and the limited area was analyzed. The high-resolution TEM image of the quasi-single crystal did not show a perfect single crystal, as supported by the SAED pattern with the mixed dot and ring patterns. The specimen of the quasi-single crystal was too vulnerable for even the relatively weak thermal energy of the room temperature, which easily dissociated the Hg_2_Br_2_ into individual molecules. Nevertheless, based on our knowledge, this is the first TEM observation of an Hg_2_Br_2_ crystal, which is a meaningful step for characterizing the van der Waals crystal with weak bonding strength.

## 4. Conclusions

In conclusion, we investigated the growth and evolution behavior of a Hg_2_Br_2_ single crystal based on simulation and experimental results. We found that the formation and growth of the seed crystal could be determined by optimizing the temperature gradient between the top and bottom heaters and by changing the ampoule position. As-synthesized single and quasi-single Hg_2_Br_2_ crystals, showing a predominant facet growth mode with the (001) plane, were characterized and compared using various analysis methods. The binding energy states and EBSD mapping images of the as-synthesized Hg_2_Br_2_ crystals were similar regardless of the kind of crystal. From the XRD analysis, the single-crystal Hg_2_Br_2_ was proved to have less lattice distortion than that of the quasi-single crystal. Furthermore, the Raman spectroscopy and cryogenic TEM analyses verified that there exist substantial differences in the atomic vibration mode and atomic structures between the two samples. Based on the corresponding results, we proposed the crystal growth modes of PVT-grown Hg_2_Br_2_, which were different depending on the growth region. This in-depth investigation of the growth mechanism for high-quality Hg_2_Br_2_ will contribute to the implementation of potentially high-performance AOTF applications.

## Supplementary Materials

The following are available online at https://www.mdpi.com/1996-1944/12/24/4224/s1: Table S1: Table summarizing peak positions of quasi-single and single Hg_2_Br_2_ crystals; Figure S1: Spectral results extracted from Raman spectroscopy mapping for (110) and (001) planes of quasi-single and single Hg_2_Br_2_ crystals. The images below show the Raman mapping images for each wavenumber; Figure S2: Scanning electron microscopy images of Cryo-FIB sample with quasi-single Hg_2_Br_2_ at (a) 63 K and (b) room temperature; (c) Low magnified and (d) high magnified HRTEM images of the quasi-single Hg_2_Br_2_ crystal. The inset of (d) shows the corresponding electron diffraction image.
