# Supplementary material for: In-depth Investigation of Hg2Br2 Crystal Growth and Evolution
Source: Materials (Basel). 2019 Dec 16;12(24):4224. doi: 10.3390/ma12244224 (PMC6947549; doi:10.3390/ma12244224)
Supplement: Supplementary file 1 [file materials-12-04224-s001.pdf]

materials

MDPI

Supplementary Materials

Table S1. Table summarizing peak positions of quasi-single and single Hg_2Br_2 crystals.

Spectrum	Peak Position			Symmetry
	Experiment		Theory [1]	
	Quasi sing Hg_2Br_2	Singel Hg_2Br_2		
Raman, cm^{-1}	-	-	35.5	E_g
	77.7	77.7	-	-
	94.9	94.9	91	E_g
	138	137.7	136	A_{1g}
	144.2	143.5	-	-
	157.8	157.8	-	-
	224.6	222.6	221	A_{1g}

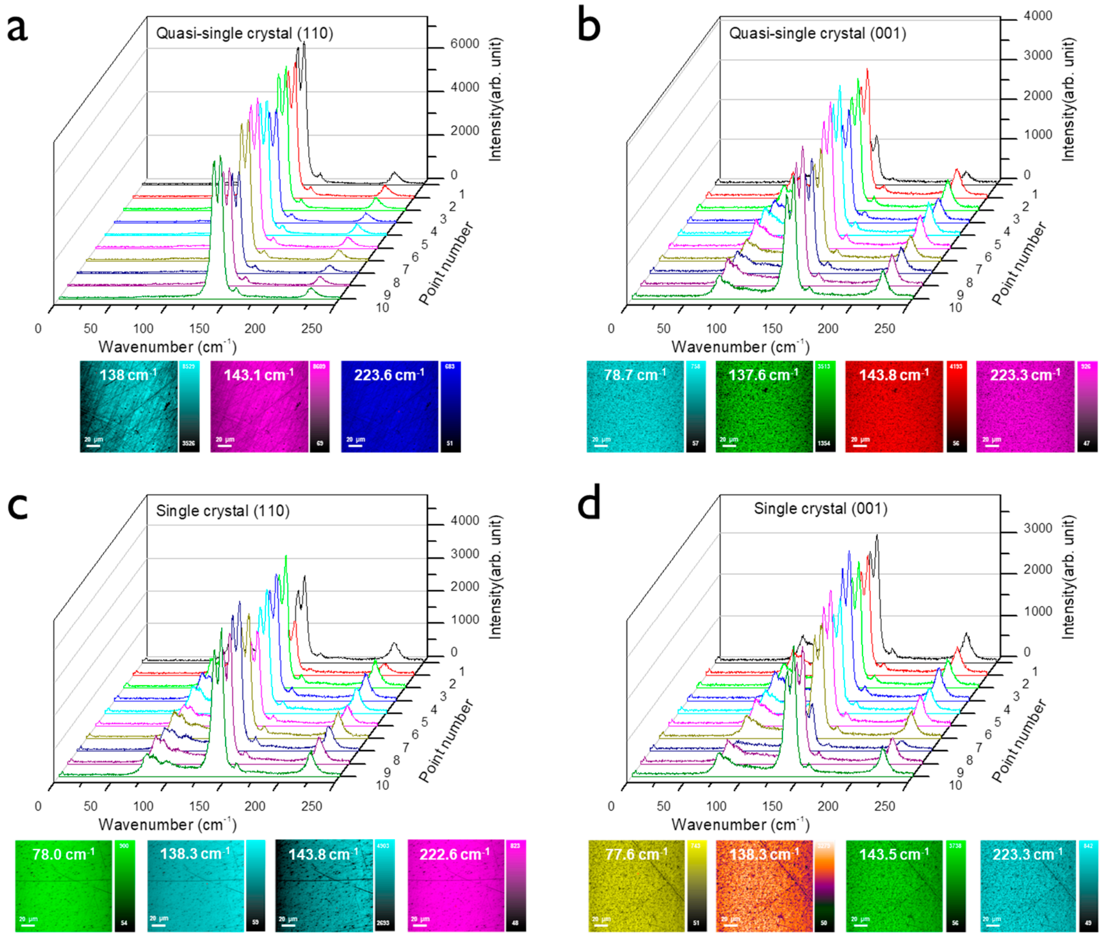

Figure S1. Spectral results extracted from Raman spectroscopy mapping for (110) and (001) planes of quasi-single and single Hg_2Br_2 crystals. The images below show the Raman mapping images for each wavenumber.

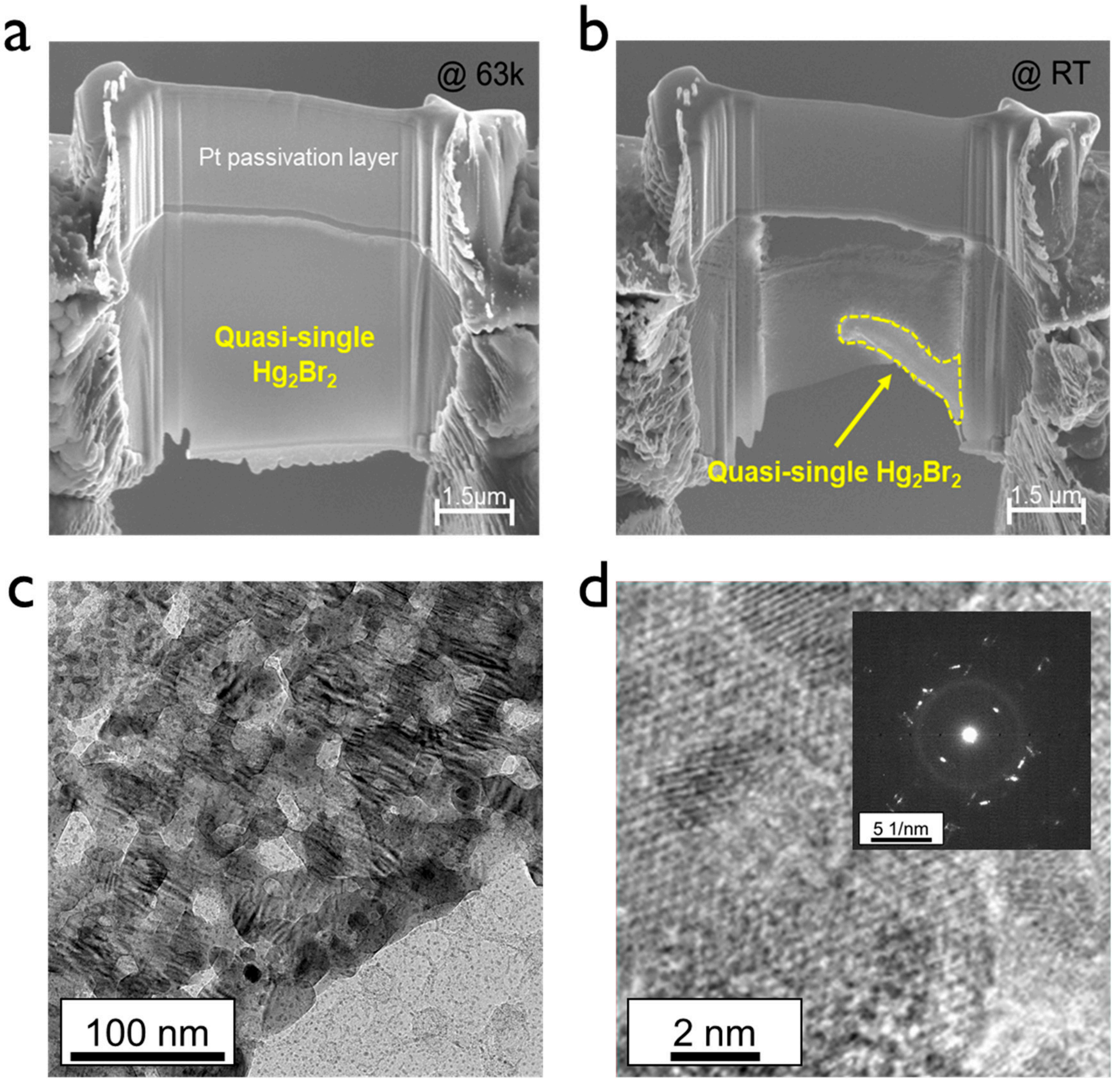

Figure S2. Scanning electron microscopy images of Cryo-FIB sample with quasi-single Hg_2Br_2 at (**a**) 63K and (**b**) room temperature; (**c**) Low magnified and (**d**) high magnified HRTEM images of the quasi-single Hg_2Br_2 crystal. The inset of (d) shows the corresponding electron diffraction image.

References

1. Roginskii, E.M.; Kvasov, A.A.; Markov, Y.F.; Smirnov, M.B. Lattice dynamics and phonon dispersion in Hg_2Br_2 model ferroelastic crystals. *Tech. Phys. Lett*. **2012**, *38*, 361.

© 2019 by the authors. Licensee MDPI, Basel, Switzerland. This article is an open access article distributed under the terms and conditions of the Creative Commons Attribution (CC BY) license (http://creativecommons.org/licenses/by/4.0/).
